# 2-Amino­benzoic acid–4,4′-bi­pyridine (2/1)

**DOI:** 10.1107/S160053681302271X

**Published:** 2013-08-17

**Authors:** Hadi D. Arman, Edward R. T. Tiekink

**Affiliations:** a Department of Chemistry, The University of Texas at San Antonio, One UTSA Circle, San Antonio, Texas 78249-0698, USA; b Department of Chemistry, University of Malaya, 50603 Kuala Lumpur, Malaysia

## Abstract

The asymmetric unit of title co-crystal, C_10_H_8_N_2_·2C_7_H_7_NO_2_, comprises a centrosymmetric 4,4′-bi­pyridine mol­ecule, and a 2-amino­benzoic acid mol­ecule in a general position. The latter is effectively planar [C—C—C—O torsion angle = 5.0 (3)°] owing to an intra­molecular N—H⋯O(carbon­yl) hydrogen bond. Three-mol­ecule aggregates are formed *via* O—H⋯N(pyrid­yl) hydrogen bonds and these are connected into supra­molecular layers in the *bc* plane by N—H⋯O(carbon­yl) hydrogen bonds and π–π inter­actions between pyridyl and benzene rings [inter-centroid distance = 3.634 (2) Å]. Layers are connected along the *a* axis by weak π–π inter­actions between benzene rings [3.964 (2) Å].

## Related literature

For co-crystals of 2-amino­benzoic acid with pyridyl derivatives, see: Arman, Kaulgud *et al.* (2012 ▶); Arman, Miller & Tiekink (2012 ▶).
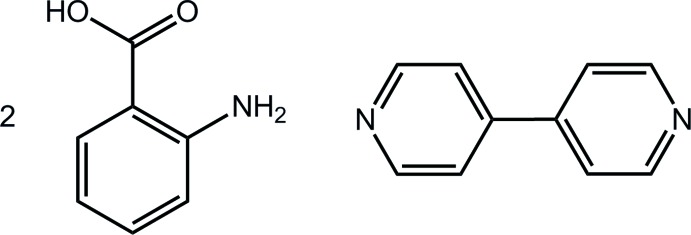


## Experimental

### 

#### Crystal data

C_10_H_8_N_2_·2C_7_H_7_NO_2_
*M* *_r_* = 430.46
Monoclinic, 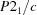

*a* = 10.782 (5) Å
*b* = 10.998 (5) Å
*c* = 8.951 (4) Å
β = 107.215 (7)°
*V* = 1013.9 (8) Å^3^
*Z* = 2
Mo *K*α radiation
μ = 0.10 mm^−1^
*T* = 98 K
0.46 × 0.11 × 0.09 mm

#### Data collection

Rigaku AFC12/SATURN724 diffractometer
Absorption correction: multi-scan (*ABSCOR*; Higashi, 1995 ▶) *T*_min_ = 0.723, *T*_max_ = 1.000
7849 measured reflections
2320 independent reflections
1932 reflections with *I* > 2σ(*I*)
*R*_int_ = 0.049
Standard reflections: 0

#### Refinement

*R*[*F* ^2^ > 2σ(*F* ^2^)] = 0.061
*wR*(*F* ^2^) = 0.159
*S* = 1.11
2320 reflections
154 parameters
4 restraints
H atoms treated by a mixture of independent and constrained refinement
Δρ_max_ = 0.32 e Å^−3^
Δρ_min_ = −0.26 e Å^−3^

### 

Data collection: *CrystalClear* (Molecular Structure Corporation & Rigaku, 2005 ▶); cell refinement: *CrystalClear*; data reduction: *CrystalClear*; program(s) used to solve structure: *SHELXS97* (Sheldrick, 2008 ▶); program(s) used to refine structure: *SHELXL97* (Sheldrick, 2008 ▶); molecular graphics: *ORTEPII* (Johnson, 1976 ▶) and *DIAMOND* (Brandenburg, 2006 ▶); software used to prepare material for publication: *publCIF* (Westrip, 2010 ▶).

## Supplementary Material

Crystal structure: contains datablock(s) general, I. DOI: 10.1107/S160053681302271X/zs2276sup1.cif

Structure factors: contains datablock(s) I. DOI: 10.1107/S160053681302271X/zs2276Isup2.hkl

Supplementary material file. DOI: 10.1107/S160053681302271X/zs2276Isup3.cml

Additional supplementary materials:  crystallographic information; 3D view; checkCIF report

## Figures and Tables

**Table 1:** Hydrogen-bond geometry (Å, °)

*D*—H⋯*A*	*D*—H	H⋯*A*	*D*⋯*A*	*D*—H⋯*A*
N1—H1n⋯O2	0.88 (1)	2.02 (2)	2.697 (2)	132 (2)
O1—H1o⋯N2^i^	0.85 (1)	1.81 (1)	2.655 (2)	174 (2)
N1—H2n⋯O2^ii^	0.88 (2)	2.14 (2)	3.002 (3)	170 (2)

Symmetry codes: (i) 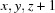; (ii) 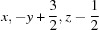.

**Fig. 1.**
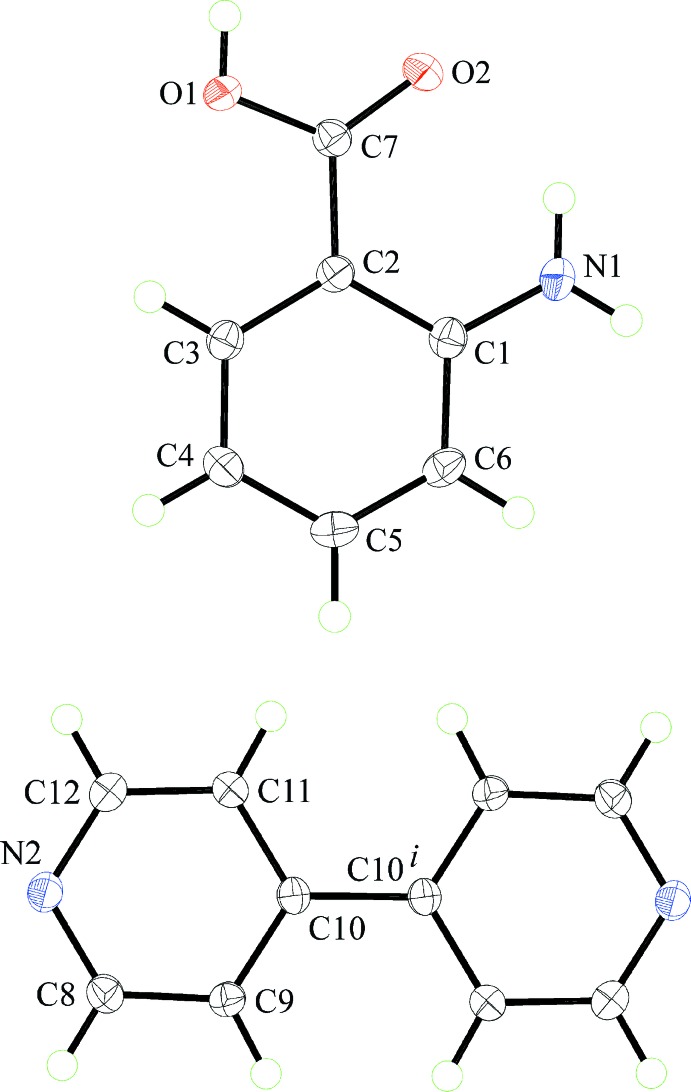
Molecular structures of the components of (I), showing atom-labelling scheme and displacement ellipsoids at the 50% probability level: (upper) 2-aminobenzoic acid and (lower) 4,4'-bipyridine. For symmetry code (i): -x + 1, -y + 1, -z + 1.

**Fig. 2.**
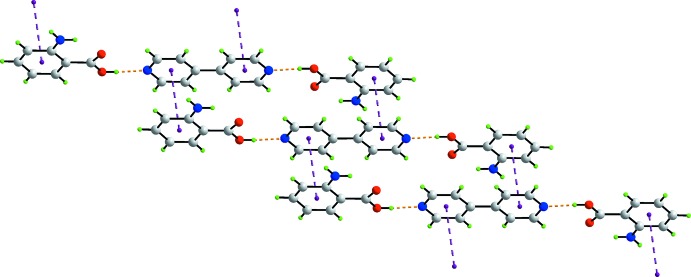
Detail of the π—π interactions between three-molecule aggregates in (I). The O—H···N and π—π interactions are shown as orange and purple dashed lines, respectively.

**Fig. 3.**
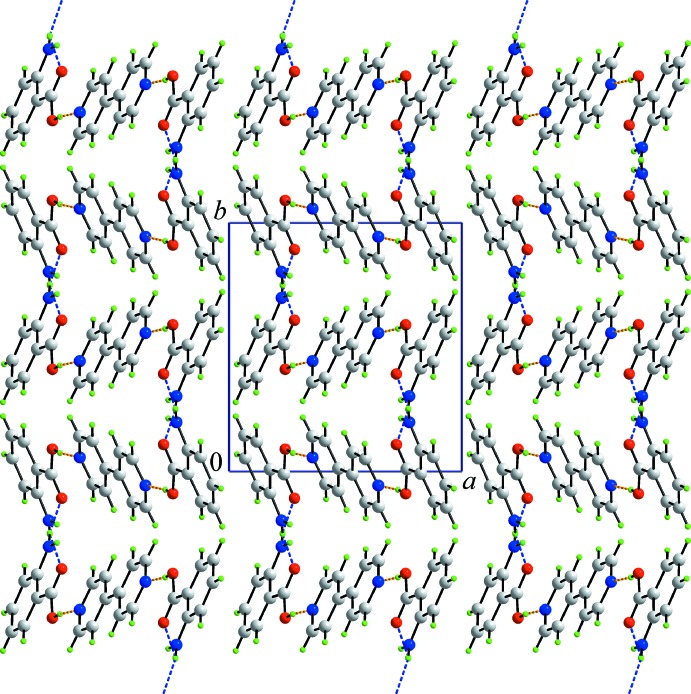
Unit-cell contents of (I) viewed in projection down the c axis. The N—H···O hydrogen bonds are shown blue dashed lines.

## supplementary crystallographic information

### 1. Comment

The title co-crystal, (I), was formed in continuation of on-going structural studies of co-crystals involving 2-aminobenzoic acid with various pyridyl derivatives (Arman, Kaulgud *et al.*, 2012; Arman, Miller & Tiekink, 2012).

The asymmetric unit contains a molecule of 2-aminobenzoic acid in a general position, and half a molecule of 4,4'-bipyridine disposed about a centre of inversion, Fig. 1. The carboxylic acid is planar owing to the presence of an intramolecular N1—H···O2 hydrogen bond, Table 1, as seen in the C1—C2—C7—O2 torsion angle of 5.0 (3)°. The acid and base associate into a centrosymmetric three-molecule aggregate *via* O1—H···N2 hydrogen bonds, Fig. 2 and Table 1. These assemble into columns along the *c* axis *via*π—π interactions between the pyridyl and benzene rings [inter-centroid distance = 3.634 (2) Å], Fig. 2. Supramolecular layers in the *bc* plane are formed by N1—H···O2 hydrogen bonds, Table 1. Connections between layers along the *a* axis are weak π—π interactions between benzene rings [inter-centroid distance = 3.964 (2) Å for symmetry operation: -*x*, - *y* + 1, -*z* + 1] (Fig. 3.)

### 2. Experimental

Crystals of (I) were obtained by the co-crystallization of 2-aminobenzoic acid (Sigma-Aldrich, 0.18 mmol) and 4,4'-bipyridine (Sigma-Aldrich, 0.14 mmol) in chloroform solution. Crystals were obtained by slow evaporation.

### 3. Refinement

C-bound H-atoms were placed in calculated positions (C—H = 0.95 Å) and were included in the refinement in the riding model approximation with *U*_iso_(H) set to 1.2*U*_eq_(C). The O- and N-bound H-atoms were located in a difference Fourier map and were refined with distance restraints of O—H = 0.84±0.01 Å and N—H = 0.88±0.01 Å, and with *U*_iso_(H) = 1.2*U*_eq_(N) and 1.5*U*_eq_(O). Owing to being affected by the beam-stop, a reflection, *i.e*. (1 0 0), was omitted from the final cycles of refinement.

### Crystal data

**Table:**

C_10_H_8_N_2_·2C_7_H_7_NO_2_	*F*(000) = 452
*M**_r_* = 430.46	*D*_x_ = 1.410 Mg m^−^^3^
Monoclinic, *P*2_1_/*c*	Mo *K*α radiation, λ = 0.71069 Å
Hall symbol: -P 2ybc	Cell parameters from 3691 reflections
*a* = 10.782 (5) Å	θ = 2.0–40.7°
*b* = 10.998 (5) Å	µ = 0.10 mm^−^^1^
*c* = 8.951 (4) Å	*T* = 98 K
β = 107.215 (7)°	Prism, yellow
*V* = 1013.9 (8) Å^3^	0.46 × 0.11 × 0.09 mm
*Z* = 2	

### Data collection

**Table:**

Rigaku AFC12K/SATURN724 diffractometer	2320 independent reflections
Radiation source: fine-focus sealed tube	1932 reflections with *I* > 2σ(*I*)
Graphite monochromator	*R*_int_ = 0.049
ω scans	θ_max_ = 27.5°, θ_min_ = 2.7°
Absorption correction: multi-scan (*ABSCOR*; Higashi, 1995)	*h* = −13→13
*T*_min_ = 0.723, *T*_max_ = 1.000	*k* = −14→14
7849 measured reflections	*l* = −11→9

### Refinement

**Table:**

Refinement on *F*^2^	Primary atom site location: structure-invariant direct methods
Least-squares matrix: full	Secondary atom site location: difference Fourier map
*R*[*F*^2^ > 2σ(*F*^2^)] = 0.061	Hydrogen site location: inferred from neighbouring sites
*wR*(*F*^2^) = 0.159	H atoms treated by a mixture of independent and constrained refinement
*S* = 1.11	*w* = 1/[σ^2^(*F*_o_^2^) + (0.0786*P*)^2^ + 0.3371*P*] where *P* = (*F*_o_^2^ + 2*F*_c_^2^)/3
2320 reflections	(Δ/σ)_max_ < 0.001
154 parameters	Δρ_max_ = 0.32 e Å^−^^3^
4 restraints	Δρ_min_ = −0.26 e Å^−^^3^

### Special details

**Table:**

Geometry. All s.u.'s (except the s.u. in the dihedral angle between two l.s. planes) are estimated using the full covariance matrix. The cell s.u.'s are taken into account individually in the estimation of s.u.'s in distances, angles and torsion angles; correlations between s.u.'s in cell parameters are only used when they are defined by crystal symmetry. An approximate (isotropic) treatment of cell s.u.'s is used for estimating s.u.'s involving l.s. planes.
Refinement. Refinement of *F*^2^ against ALL reflections. The weighted *R*-factor *wR* and goodness of fit *S* are based on *F*^2^, conventional *R*-factors *R* are based on *F*, with *F* set to zero for negative *F*^2^. The threshold expression of *F*^2^ > 2σ(*F*^2^) is used only for calculating *R*-factors(gt) *etc*. and is not relevant to the choice of reflections for refinement. *R*-factors based on *F*^2^ are statistically about twice as large as those based on *F*, and *R*- factors based on ALL data will be even larger.

### Fractional atomic coordinates and isotropic or equivalent isotropic displacement parameters (Å^2^)

**Table:**

	*x*	*y*	*z*	*U*_iso_*/*U*_eq_	
O1	0.24135 (13)	0.41046 (12)	0.79067 (14)	0.0227 (3)	
H1O	0.277 (2)	0.426 (2)	0.8863 (13)	0.034*	
O2	0.28109 (13)	0.60804 (12)	0.76583 (14)	0.0227 (3)	
N1	0.22588 (18)	0.69761 (15)	0.47292 (19)	0.0275 (4)	
H1N	0.264 (2)	0.711 (2)	0.5733 (11)	0.041*	
H2N	0.233 (2)	0.7512 (18)	0.4034 (19)	0.041*	
N2	0.35899 (15)	0.44351 (14)	0.09341 (17)	0.0204 (4)	
C1	0.17630 (16)	0.58551 (17)	0.4277 (2)	0.0190 (4)	
C2	0.17652 (16)	0.49167 (16)	0.53666 (19)	0.0174 (4)	
C3	0.11939 (16)	0.37885 (17)	0.4823 (2)	0.0185 (4)	
H3	0.1171	0.3173	0.5558	0.022*	
C4	0.06660 (17)	0.35534 (18)	0.3247 (2)	0.0216 (4)	
H4	0.0291	0.2784	0.2897	0.026*	
C5	0.06951 (17)	0.44668 (19)	0.2180 (2)	0.0225 (4)	
H5	0.0343	0.4312	0.1092	0.027*	
C6	0.12234 (18)	0.55891 (17)	0.2671 (2)	0.0211 (4)	
H6	0.1225	0.6196	0.1916	0.025*	
C7	0.23616 (16)	0.51026 (16)	0.7065 (2)	0.0183 (4)	
C8	0.35778 (17)	0.35879 (17)	0.2006 (2)	0.0209 (4)	
H8	0.3171	0.2831	0.1659	0.025*	
C9	0.41319 (17)	0.37652 (16)	0.3599 (2)	0.0190 (4)	
H9	0.4122	0.3129	0.4313	0.023*	
C10	0.47043 (16)	0.48801 (16)	0.41530 (18)	0.0163 (4)	
C11	0.46902 (18)	0.57685 (17)	0.3028 (2)	0.0210 (4)	
H11	0.5050	0.6549	0.3341	0.025*	
C12	0.41485 (17)	0.55033 (18)	0.1457 (2)	0.0225 (4)	
H12	0.4174	0.6110	0.0712	0.027*	

### Atomic displacement parameters (Å^2^)

**Table:**

	*U*^11^	*U*^22^	*U*^33^	*U*^12^	*U*^13^	*U*^23^
O1	0.0326 (7)	0.0183 (7)	0.0149 (6)	−0.0019 (6)	0.0036 (5)	0.0017 (5)
O2	0.0302 (7)	0.0181 (7)	0.0193 (6)	−0.0021 (6)	0.0065 (5)	−0.0038 (5)
N1	0.0423 (10)	0.0171 (8)	0.0225 (8)	−0.0046 (7)	0.0087 (7)	0.0027 (6)
N2	0.0229 (7)	0.0210 (8)	0.0167 (7)	0.0022 (6)	0.0048 (6)	0.0001 (6)
C1	0.0198 (8)	0.0165 (9)	0.0216 (9)	0.0025 (7)	0.0076 (7)	0.0007 (7)
C2	0.0198 (8)	0.0162 (9)	0.0167 (8)	0.0026 (7)	0.0064 (7)	0.0002 (6)
C3	0.0193 (8)	0.0168 (9)	0.0197 (8)	0.0015 (7)	0.0061 (7)	0.0015 (6)
C4	0.0211 (8)	0.0195 (9)	0.0227 (9)	−0.0018 (7)	0.0043 (7)	−0.0028 (7)
C5	0.0219 (9)	0.0276 (10)	0.0164 (8)	0.0031 (8)	0.0030 (7)	−0.0002 (7)
C6	0.0246 (9)	0.0213 (9)	0.0176 (8)	0.0056 (7)	0.0066 (7)	0.0037 (7)
C7	0.0198 (8)	0.0173 (9)	0.0185 (8)	0.0013 (7)	0.0065 (7)	−0.0016 (6)
C8	0.0239 (9)	0.0186 (9)	0.0197 (8)	0.0004 (7)	0.0059 (7)	−0.0024 (7)
C9	0.0227 (8)	0.0163 (9)	0.0179 (8)	0.0010 (7)	0.0056 (7)	0.0011 (6)
C10	0.0155 (8)	0.0182 (9)	0.0156 (8)	0.0009 (7)	0.0053 (6)	−0.0015 (6)
C11	0.0254 (9)	0.0186 (9)	0.0185 (8)	−0.0040 (7)	0.0054 (7)	−0.0012 (7)
C12	0.0259 (9)	0.0226 (9)	0.0184 (8)	−0.0008 (8)	0.0054 (7)	0.0026 (7)

### Geometric parameters (Å, º)

**Table:**

O1—C7	1.323 (2)	C4—C5	1.393 (3)
O1—H1O	0.845 (10)	C4—H4	0.9500
O2—C7	1.233 (2)	C5—C6	1.376 (3)
N1—C1	1.357 (2)	C5—H5	0.9500
N1—H1N	0.882 (9)	C6—H6	0.9500
N1—H2N	0.876 (9)	C8—C9	1.387 (2)
N2—C12	1.340 (3)	C8—H8	0.9500
N2—C8	1.341 (2)	C9—C10	1.396 (3)
C1—C6	1.412 (2)	C9—H9	0.9500
C1—C2	1.420 (2)	C10—C11	1.400 (2)
C2—C3	1.407 (3)	C10—C10^i^	1.484 (3)
C2—C7	1.479 (2)	C11—C12	1.385 (2)
C3—C4	1.380 (2)	C11—H11	0.9500
C3—H3	0.9500	C12—H12	0.9500
			
C7—O1—H1O	109.8 (17)	C5—C6—H6	119.4
C1—N1—H1N	118.5 (15)	C1—C6—H6	119.4
C1—N1—H2N	120.5 (15)	O2—C7—O1	122.21 (16)
H1N—N1—H2N	120.1 (16)	O2—C7—C2	123.99 (16)
C12—N2—C8	117.25 (15)	O1—C7—C2	113.76 (15)
N1—C1—C6	119.95 (16)	N2—C8—C9	123.08 (17)
N1—C1—C2	122.33 (16)	N2—C8—H8	118.5
C6—C1—C2	117.72 (17)	C9—C8—H8	118.5
C3—C2—C1	119.46 (15)	C8—C9—C10	119.95 (16)
C3—C2—C7	119.36 (15)	C8—C9—H9	120.0
C1—C2—C7	121.18 (16)	C10—C9—H9	120.0
C4—C3—C2	121.65 (16)	C9—C10—C11	116.62 (16)
C4—C3—H3	119.2	C9—C10—C10^i^	122.0 (2)
C2—C3—H3	119.2	C11—C10—C10^i^	121.4 (2)
C3—C4—C5	118.65 (18)	C12—C11—C10	119.66 (17)
C3—C4—H4	120.7	C12—C11—H11	120.2
C5—C4—H4	120.7	C10—C11—H11	120.2
C6—C5—C4	121.28 (17)	N2—C12—C11	123.39 (17)
C6—C5—H5	119.4	N2—C12—H12	118.3
C4—C5—H5	119.4	C11—C12—H12	118.3
C5—C6—C1	121.18 (16)		
			
N1—C1—C2—C3	177.74 (16)	C1—C2—C7—O2	5.0 (3)
C6—C1—C2—C3	−2.4 (2)	C3—C2—C7—O1	6.9 (2)
N1—C1—C2—C7	−2.5 (3)	C1—C2—C7—O1	−172.86 (16)
C6—C1—C2—C7	177.36 (15)	C12—N2—C8—C9	−1.2 (3)
C1—C2—C3—C4	2.2 (3)	N2—C8—C9—C10	1.9 (3)
C7—C2—C3—C4	−177.63 (16)	C8—C9—C10—C11	−0.5 (3)
C2—C3—C4—C5	−0.6 (3)	C8—C9—C10—C10^i^	179.24 (19)
C3—C4—C5—C6	−0.7 (3)	C9—C10—C11—C12	−1.3 (3)
C4—C5—C6—C1	0.3 (3)	C10^i^—C10—C11—C12	178.92 (19)
N1—C1—C6—C5	−178.94 (17)	C8—N2—C12—C11	−0.7 (3)
C2—C1—C6—C5	1.2 (3)	C10—C11—C12—N2	2.0 (3)
C3—C2—C7—O2	−175.22 (16)		

Symmetry code: (i) −*x*+1, −*y*+1, −*z*+1.

### Hydrogen-bond geometry (Å, º)

**Table:**

*D*—H···*A*	*D*—H	H···*A*	*D*···*A*	*D*—H···*A*
N1—H1n···O2	0.88 (1)	2.02 (2)	2.697 (2)	132 (2)
O1—H1o···N2^ii^	0.85 (1)	1.81 (1)	2.655 (2)	174 (2)
N1—H2n···O2^iii^	0.88 (2)	2.14 (2)	3.002 (3)	170 (2)

Symmetry codes: (ii) *x*, *y*, *z*+1; (iii) *x*, −*y*+3/2, *z*−1/2.
